# Immune Checkpoint Inhibitor Therapy Aggravates T Cell–Driven Plaque Inflammation in Atherosclerosis

**DOI:** 10.1016/j.jaccao.2020.08.007

**Published:** 2020-10-06

**Authors:** Kikkie Poels, Mandy M.T. van Leent, Celine Boutros, Hubert Tissot, Séverine Roy, Anu E. Meerwaldt, Yohana C.A. Toner, Myrthe E. Reiche, Pascal J.H. Kusters, Tsveta Malinova, Stephan Huveneers, Audrey E. Kaufman, Venkatesh Mani, Zahi A. Fayad, Menno P.J. de Winther, Aurelien Marabelle, Willem J.M. Mulder, Caroline Robert, Tom T.P. Seijkens, Esther Lutgens

**Affiliations:** a Department of Medical Biochemistry, Amsterdam Cardiovascular Sciences, Amsterdam University Medical Centers, University of Amsterdam, Amsterdam, the Netherlands; b Biomedical Engineering and Imaging Institute, Icahn School of Medicine at Mount Sinai, New York, New York, USA; c Oncology Department, Institut de Cancérologie Gustave Roussy, Villejuif, France; d Radiology Department, Institut de Cancérologie Gustave Roussy, Paris, France; e Biomedical MR Imaging and Spectroscopy Group, Center for Image Sciences, University Medical Center Utrecht, Utrecht University, Utrecht, the Netherlands; f Laboratory of Chemical Biology, Department of Biomedical Engineering and Institute for Complex Molecular Systems, Eindhoven University of Technology, Eindhoven, the Netherlands; g Faculty of Medicine, Universite Paris-Saclay, Le Kremlin-Bicêtre, France; h Department of Hematology, Amsterdam University Medical Centers, Vrije Universiteit Amsterdam, Amsterdam, the Netherlands; i Institute for Cardiovascular Prevention, Ludwig Maximilian University of Munich, Munich, Germany; j German Centre for Cardiovascular Research, partner site Munich Heart Alliance, Munich, Germany

**Keywords:** atherosclerosis, CTLA4, immune checkpoint inhibitors, inflammation, PD-1, ^18^F-FDG, 2-deoxy-2-[fluorine-18]fluoro-D-glucose, CT, computed tomography, CVD, cardiovascular disease, FCA, fibrous cap atheroma, ICAM, intercellular adhesion molecule, ICI, immune checkpoint inhibitor, PET, positron emission tomography, VCAM, vascular cell adhesion molecule

## Abstract

**Background:**

Immunotherapy has revolutionized cancer treatment. However, immune checkpoint inhibitors (ICIs) that target PD-1 (programmed cell death protein-1) and/or CTLA-4 (cytotoxic T lymphocyte-associated antigen-4) are commonly associated with acute immune-related adverse events. Accumulating evidence also suggests that ICIs aggravate existing inflammatory diseases.

**Objectives:**

As inflammation drives atherosclerotic cardiovascular disease, we studied the propensity of short-term ICI therapy to aggravate atherosclerosis.

**Methods:**

We used ^18^F-FDG (2-deoxy-2-[fluorine-18]fluoro-D-glucose) positron emission tomography–computed tomography to detect macrophage-driven vascular and systemic inflammation in pembrolizumab and nivolumab/ipilimumab–treated melanoma patients. In parallel, atherosclerotic *Ldlr*^*–/–*^ mice were treated with CTLA-4 and PD-1 inhibition to study the proinflammatory consequences of immune checkpoint inhibition.

**Results:**

ICI treatment did not affect ^18^F-FDG uptake in the large arteries, spleen, and bone marrow of melanoma patients, nor myeloid cell activation in blood and lymphoid organs in hyperlipidemic mice. In contrast, we found marked changes in the adaptive immune response (i.e., increased CD4^+^ effector T cell and CD8^+^ cytotoxic T cell numbers in lymphoid organs and the arterial wall of our hyperlipidemic mice). Although plaque size was unaffected, plaques had progressed toward a lymphoid-based inflammatory phenotype, characterized by a 2.7-fold increase of CD8^+^ T cells and a 3.9-fold increase in necrotic core size. Increased endothelial activation was observed with a 2.2-fold and 1.6-fold increase in vascular cell adhesion molecule-1 and intercellular adhesion molecule-1, respectively.

**Conclusions:**

This study demonstrates that combination therapy with anti-CTLA-4 and anti-PD-1 antibodies does not affect myeloid-driven vascular and systemic inflammation in melanoma patients and hyperlipidemic mice. However, short-term ICI therapy in mice induces T cell–mediated plaque inflammation and drives plaque progression.

Immune checkpoint inhibitors (ICIs) are monoclonal antibodies that antagonize co-inhibitory molecules, such as CTLA-4 (cytotoxic T lymphocyte-associated antigen 4), PD-1 (programmed cell death protein-1) or PD-L1 (programmed cell death protein ligand-1) (1,2). They release the natural brake on T cell activation, which elicits potent antitumor immune responses (3,4). ICIs have revolutionized cancer immunotherapy and have shown efficacy in the treatment of many types of cancer, including melanoma and non-small cell lung cancer, and are considered standard of care for these and other malignancies (1,5,6). Combination strategies that target both CTLA-4 and PD-1 are increasingly applied in the clinic (7,8).

Unfortunately, immune-related adverse events are a common side effect of immune checkpoint inhibition and are caused by T cell–mediated cytotoxicity in various organs, with skin, intestine, lungs, liver, and endocrine organs being affected most frequently (2,7,9). Up to 59% of the patients who receive combination CTLA-4 and PD-1 inhibitors develop adverse events that are considered severe (2,10). Most of these events can be successfully overcome by corticosteroids treatment and (temporary) discontinuation of immune therapy (2,10). Recent evidence reveals that ICI therapy also aggravates pre-existing autoimmune and chronic inflammatory diseases in cancer patients: 27% to 75% of cancer patients with a history of rheumatoid arthritis, systemic lupus erythematosus, or inflammatory bowel disease experience an exacerbation upon ICI treatment (11,12).

A concern of ICI therapy is the potential adverse effects on the progression of cardiovascular disease (CVD) (13,14). Atherosclerosis, the underlying pathology of many cardiovascular events, is a chronic inflammatory disease of the larger arteries. Both lymphoid and myeloid immune cells drive atherosclerotic plaque formation and progression toward clinically unfavorable, unstable lesions that may cause myocardial infarction or ischemic stroke (15). Whether immune checkpoint inhibition affects the inflammatory process that underlies atherosclerosis is currently unknown. However, ICI-related atherosclerotic CVD is increasingly reported in clinical studies and case reports (16, 17, 18).

In this study, we investigated the effects of short-term ICI therapy on vascular inflammation in a small group of patients with stage IV melanoma and in hyperlipidemic mice using a combination of 2-deoxy-2-[fluorine-18]fluoro-D-glucose (^18^F-FDG) positron emission tomography–computed tomography (PET/CT) and immunological techniques.

## Methods

### Patient characteristics

Patient characteristics are shown in Table 1. Ten patients with stage IV melanoma were treated in first line with pembrolizumab (2 mg/kg) every 3 weeks (n = 1) or the combination of nivolumab (1 mg/kg) and ipilimumab (n = 9) every 4 weeks. Ipilimumab was administered intravenously (3 mg/kg; n = 2) or intratumorally (0.3 mg/kg; n = 7) in a clinical trial setting (NCT02857569). Approval for use of the patient data was provided by the ethical committee of Le Kremlin-Bicêtre in France (permit number MSN #2008-A00373-52).

**Table 1:** Characteristics of Stage IV Melanoma Patients

ID	Sex	Age (yrs)	ICI Therapy	Cardiovascular Risk Factors
1	M	56	Ipilimumab (IT), nivolumab (intravenous)	None
2	M	62	Ipilimumab (IV), nivolumab (intravenous)	None
3	F	54	Ipilimumab (IT), nivolumab (intravenous)	Hypertension
4	F	46	Ipilimumab (IV), nivolumab (intravenous)	None
5	F	57	Ipilimumab (IT), nivolumab (intravenous)	None
6	M	37	Ipilimumab (IT), nivolumab (intravenous)	Hypertension
7	M	65	Ipilimumab (IT), nivolumab (intravenous)	Hypertension, dyslipidemia
8	F	52	Ipilimumab (IT), nivolumab (intravenous)	None
9	M	48	Ipilimumab (IT), nivolumab (intravenous)	Tobacco smoking
10	F	50	Pembrolizumab	Hypertension

IT = intratumoral.

### ^18^F-FDG PET/CT imaging and analysis

^18^F-FDG PET/CT imaging was performed in humans and atherosclerotic *Apoe*^*–/–*^ mice (Supplemental Appendix). Image analysis of the human PET/CT data was performed by an experienced radiologist (A.E.K.) using OsiriX MD version 8.0.2 (Pixmeo, Geneva, Switzerland). CT images extending from the level of the carotid artery bifurcation in the neck through the level of the distal femur were fused with ^18^F-FDG PET images of the same regions and analyzed in the axial plane. The technique employed has been previously described (19,20). Expanded methods, including the ^18^F--FDG PET/CT imaging of the atherosclerotic *Apoe*^*–/–*^ mice, are available in the Supplemental Appendix.

### Histology

At 17 weeks of age, *Ldlr*^*–/–*^ mice were sacrificed, and the arterial tree was perfused with phosphate-buffered saline and 1% paraformaldehyde. The aortic root and aortic arch were isolated and fixed in 1% paraformaldehyde overnight. Longitudinal sections (4 μm) of the aortic arch and aortic root were stained with hematoxylin and eosin and analyzed for plaque extent, phenotype, and necrotic core size, as described previously (21). Intimal xanthoma, pathological intimal thickening, and fibrous cap atheroma (FCA) were identified (22). Expanded methods are available in the Supplemental Appendix.

### Statistics

Data are depicted as mean ± SEM. Normal distribution was analyzed with a D’Agostino-Pearson omnibus normality test, while between group differences were compared using an unpaired Student *t* test or Mann-Whitney test as appropriate based on variable distribution. Paired *t* tests were used to analyze differences in patient ^18^F-FDG PET signal at baseline and post-treatment. A chi-square test was used to analyze the contingency of the Virmani classifications. For all analyses, GraphPad Prism 5.0 software (GraphPad Software, San Diego, California) was used. All p values <0.05 were considered significant.

## Results

### Short-term ICI therapy does not affect ^18^F-FDG uptake in the large arteries, spleen, or bone marrow of melanoma patients

**Figure 1:**
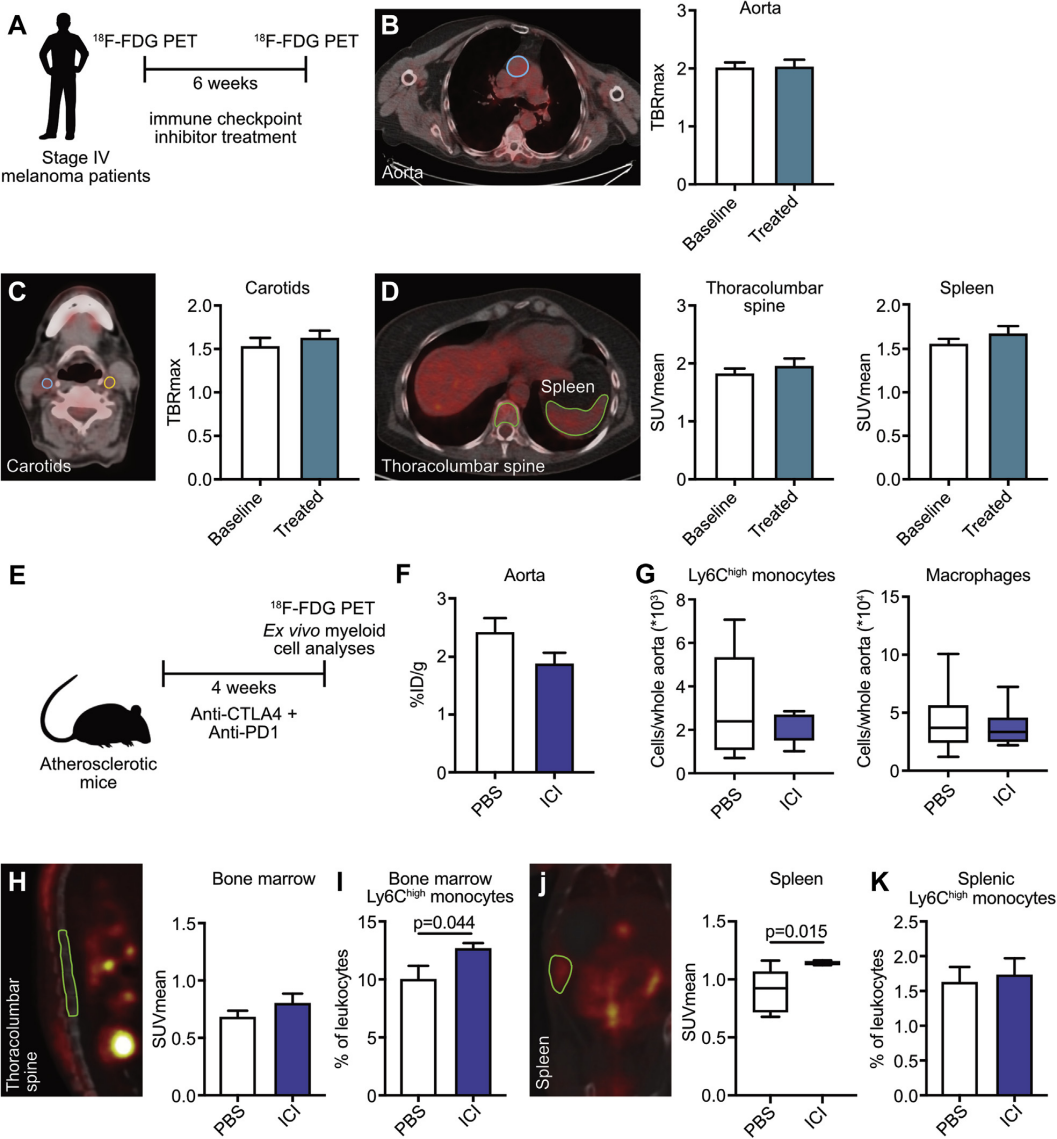
^18^F-FDG PET–Based Analysis of ICI Therapy in Humans and *Apoe*^*–/–*^ Mice **(A to D)** Ten stage IV melanoma patients underwent 2-deoxy-2-[fluorine-18]fluoro-D-glucose (^18^F-FDG) positron emission tomography–computed tomography (PET/CT) imaging at baseline and 6 weeks after the start of immune checkpoint inhibitor (ICI) therapy. **(A)** Schematic overview of experimental design. **(B)** Representative PET/CT image and maximum target-to-background ratio (TBRmax) of the thoracic aorta before (baseline) and after ICI therapy (treated). **(C)** Representative PET/CT image and TBRmax of common carotid arteries before and after ICI therapy. **(D)** Representative PET/CT image and mean standardized uptake value (SUVmean) of the thoracolumbar spine and the spleen, before and after ICI therapy. **(E)** Schematic overview of experimental design. *Apoe*^*–/–*^ mice (n = 8) were treated with anti-CTLA-4 (anti-cytotoxic T lymphocyte-associated antigen-4)/anti-PD-1 (anti-programmed cell death protein-1) antibodies or phosphate-buffered saline (PBS) for 4 weeks and subjected to ^18^F-FDG PET/CT imaging. **(F)** Ex vivo quantification of ^18^F-FDG accumulation in the aorta. **(G)** Flow cytometry analysis of Ly6C^hight^ monocytes and macrophages in the aorta. **(H)** Representative PET/CT image and SUVmean of the thoracolumbar spine. **(I)** Flow cytometry analysis of Ly6C^high^ monocytes in the bone marrow. **(J)** Representative PET/CT image and SUVmean of the spleen. **(K)** Flow cytometry analysis of Ly6C^high^ monocytes in the spleen. For all graphs, bars represent mean ± SEM.

We analyzed ^18^F-FDG PET/CT scans from 10 patients with stage IV melanoma who received pembrolizumab (2 mg/kg) every 3 weeks (n = 1) or the combination of nivolumab (1 mg/kg) and ipilimumab (n = 9) (Table 1, Figure 1A). None of the patients had a history of CVD. Cardiovascular risk factors were present in 5 of 10 patients (Table 1). ^18^F-FDG uptake in the thoracic aorta (Figure 1B) and carotid arteries (Figure 1C), as well as in spleen and bone marrow, was determined before and 6 weeks after the initiation of immune checkpoint inhibition. After 6 weeks of treatment, ^18^F-FDG uptake had not increased in the thoracic aorta, carotid arteries, or spleen or bone marrow. In this small human cohort, these data suggest that short-term ICI therapy does not affect vascular or systemic inflammation (Figure 1D).

### Anti-CTLA-4/anti-PD-1 treatment does not affect monocyte/macrophage-driven inflammation in hyperlipidemic mice

In a similar setting, we determined ^18^F-FDG uptake in the aortas, spleen, and bone marrow of 15 week old mice *Apoe*^*–/–*^ mice that had been treated twice a week with anti-CTLA-4 and anti-PD-1 antibodies for 4 weeks (Figures 1E and 1K). Similar to our observations in patients, ICI treatment did not affect ^18^F-FDG uptake in the aorta compared with control animals treated with phosphate-buffered saline (Figure 1F). Flow cytometry of aortic lysates showed no significant changes in Ly6C^high^ monocyte, Ly6C^low^ monocyte, macrophage, and neutrophil content in the aorta (Figure 1G, Supplemental Figure 1A). A slight increase in Ly6C^high^ monocytes in the bone marrow of the treated group was observed (p = 0.044), but in accordance with the patient data, no difference in ^18^F-FDG uptake could be detected in bone marrow compared with control animals (Figures 1H and 1I, Supplemental Figure 1B). Conversely, there was a slight increase in ^18^F-FDG uptake in the spleen after ICI treatment (mean standardized uptake value: control 0.910 ± 0.063 vs. ICI 1.139 ± 0.006; p = 0.015), without an effect on the Ly6C^high^ monocyte numbers. (Figures 1J and 1K, Supplemental Figure 1B). These data show that short-term ICI treatment does not have a major effect on monocyte- or macrophage-driven vascular inflammation in hyperlipidemic mice as compared with control animals.

### Immune checkpoint inhibition causes an effector T cell profile in atherosclerotic mice

**Figure 2:**
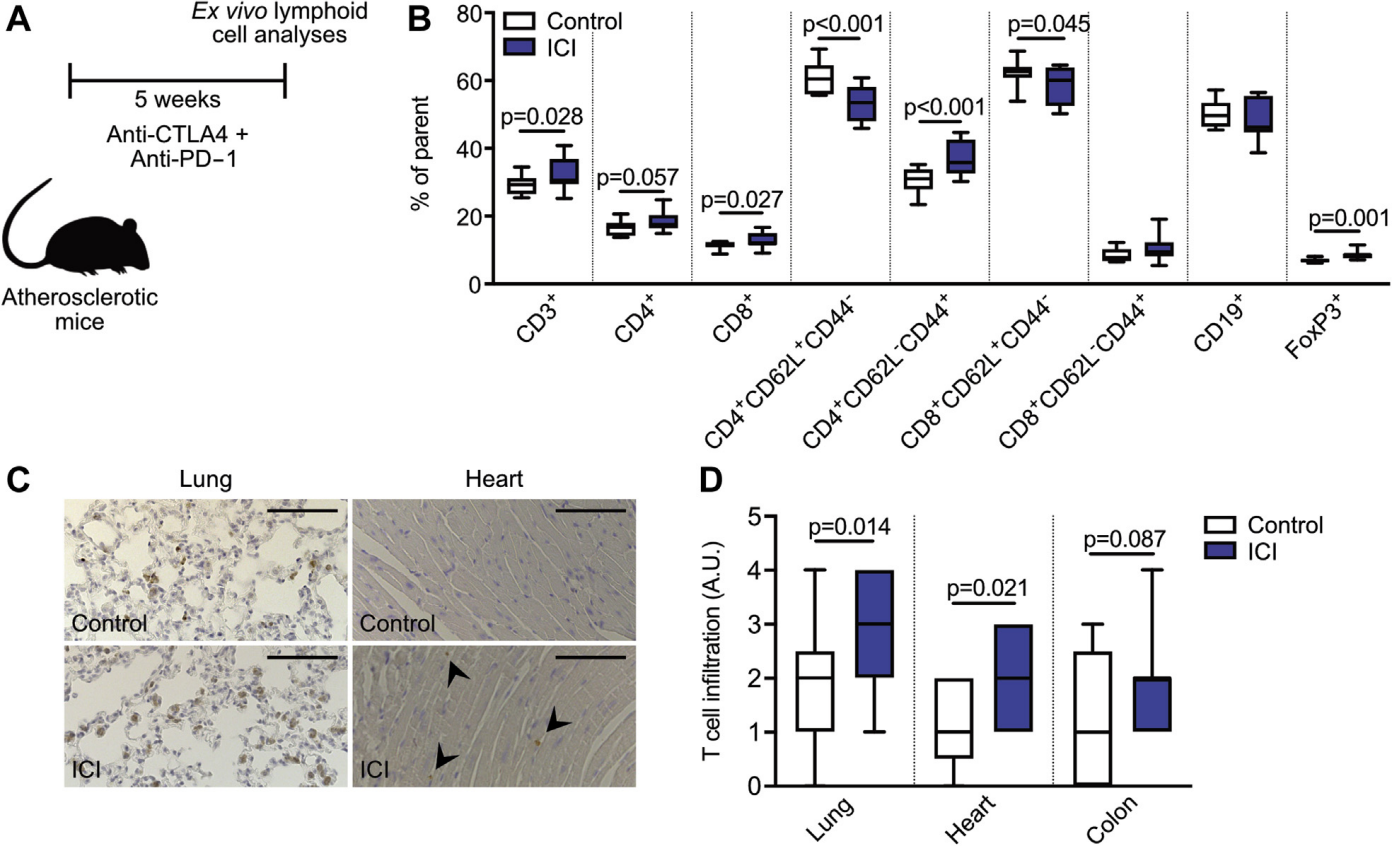
ICIs Induce a Systemic Activated T Cell Profile in *Ldlr*^*–/–*^ Mice Male *Ldlr*^*–/–*^ mice first received 6 weeks of 0.15% high cholesterol diet, after which they were treated twice a week with αCTLA-4 and αPD-1 antibodies (n = 15) or isotype control (n = 14) for 5 weeks. T cell analysis was performed on various tissues. **(A)** Schematic of experimental design. **(B)** Flow cytometric analyses of the spleen show an increase of CD4^+^ and CD8^+^ cells, as well as a shift from naive to effector/memory cells in treated mice. **(C)** Representative images of CD3 staining in the lung **(left)** and heart **(right)**, of control and ICI-treated mice. Scale bars represent 100 μm. **(D)** Histological analyses of CD3^+^ cells in the lung, heart, and colon. ICI treatment increased T cell infiltration in all 3 tissues. For all graphs, bars represent mean ± SEM. A.U. = arbitrary units; other abbreviations as in Figure 1.

To investigate the effects of immune checkpoint inhibition on atherosclerosis in more detail, we treated 12-week-old *Ldlr*^*–/–*^ mice on a 0.15% cholesterol diet twice a week with anti-CTLA4 and anti-PD1 antibodies for 5 weeks (Figure 2A). ICI treatment had no effect on the amount or subset composition of splenic B cells, myeloid, and dendritic cells (Figure 2B, Supplemental Figure 2A). In contrast, at 5 weeks, compared with control, the T cell population underwent major changes with treatment. Upon short-term anti-CTLA-4/anti-PD-1 treatment, splenic CD3^+^CD8^+^ cytotoxic T cells as well as CD3^+^CD4^+^ helper T cells increased (Figure 2B). Effector-memory (CD62L^–^CD44^+^) CD4^+^ T cells in particular increased (% parent: control 30.41 ± 0.97 vs. ICI 37.16 ± 1.45; p < 0.001), whereas naive (CD62L^+^CD44^–^) CD4^+^ and CD8^+^ T cells declined (CD4 % parent: control 60.77 ± 1.21 vs. ICI 53.46 ± 1.49; p < 0.001; CD8 % parent: control 62.50 ± 1.01 vs. ICI 58.68 ± 1.56; p = 0.045) (Figure 2B), reflective of an activated T cell profile. In parallel, splenic regulatory T cells expanded (% parent: control 6.89 ± 0.19 vs. ICI 8.65 ± 0.53; p = 0.001), possibly to compensate for the increased numbers of effector memory T cells (Figure 2B). A similarly activated T cell profile occurred in blood (Supplemental Figure 2B). In accordance with the systemic increase of T cell activation, ICI treatment caused increased T cell infiltration in nonhematopoietic organs, in particular in the lungs, heart, and colon (Figures 2C and 2D). These data demonstrate that short-term ICI treatment induces an activated T cell profile in hyperlipidemic mice, without directly affecting the myeloid system.

### ICI therapy promotes the progression of atherosclerosis and aggravates plaque inflammation

**Figure 3:**
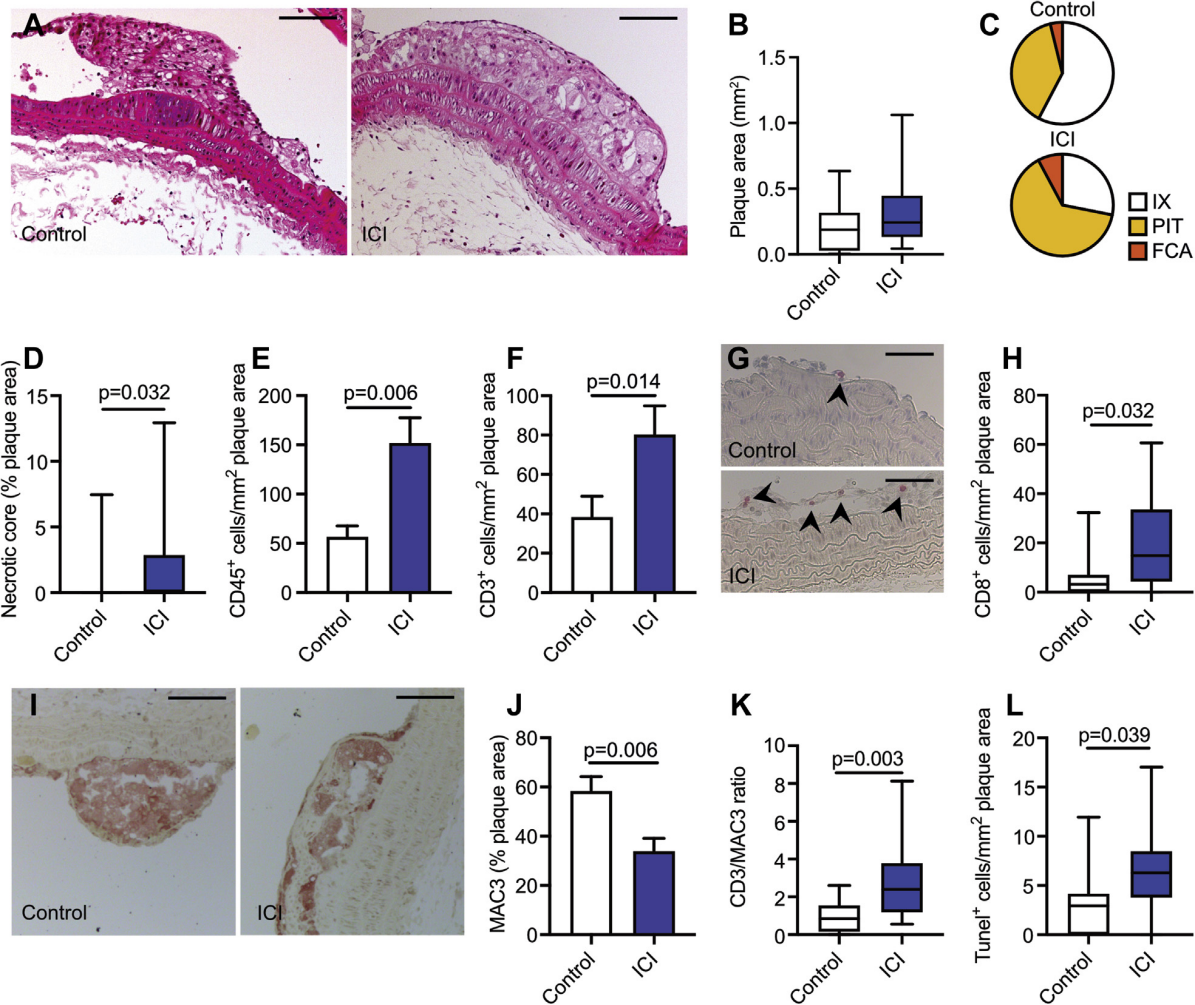
Effects of ICI Treatment on Atherosclerosis Male *Ldlr*^*–/–*^ mice on a high-cholesterol diet were treated twice a week with anti-CTLA-4 and anti-PD-1 antibodies (n = 15) or isotype control (n = 14) for 5 weeks. The aortic arch was analyzed. **(A)** Representative hematoxylin and eosin images of the aortic arch of control and ICI-treated mice. Scale bars represent 100 μm. **(B)** Analysis of plaque size in the aortic arch. **(C)** Virmani classification of aortic plaques from control and ICI-treated mice. In the latter group, fibrous cap atheromas (FCAs) and pathological intimal thickening (PIT) increased. **(D)** Quantification of necrotic core area as a percentage of plaque area. Histologic quantification of **(E)** CD45^+^ and **(F)** CD3^+^ cells in the aortic plaque. **(G)** Representative images of aortic arch CD8^+^ staining in control and ICI-treated mice. Scale bars represent 50 μm. **(H)** Quantification of CD8^+^ cells in the plaque. **(I)** Representative images of aortic arch MAC3 staining in control and ICI-treated mice. Scale bars represent 100 μm. **(J)** Analysis of MAC3-positive areas, represented as a percentage of total plaque area. **(K)** Ratio of CD3-positive to MAC3-positive areas in the aortic arch. **(L)** Histologic analysis of TUNEL^+^ (terminal deoxynucleotidyl transferase dUTP nick end labeling positive) cells in the aortic plaque. For all graphs, bars represent mean ± SEM. IX = intimal xanthoma; other abbreviations as in Figure 1.

Analysis of atherosclerotic lesion size in the aortic arch with its main branch points shows that short-term anti-CTLA-4/anti-PD-1 treatment had no effect on plaque area per se (Figures 3A and 3B). However, and more importantly, immune checkpoint inhibition did cause more advanced atherosclerosis. Morphological characterization of the atherosclerotic plaque phenotype demonstrated that the aortic arch of anti-CTLA-4/anti-PD-1–treated mice contained more pathological intimal thickening and FCAs than control mice (Figure 3C, Supplemental Figure 3A). In accordance with this more advanced plaque phenotype, a 3.9-fold increase in plaque necrotic core area was observed upon ICI treatment (Figure 3D).

**Figure 4:**
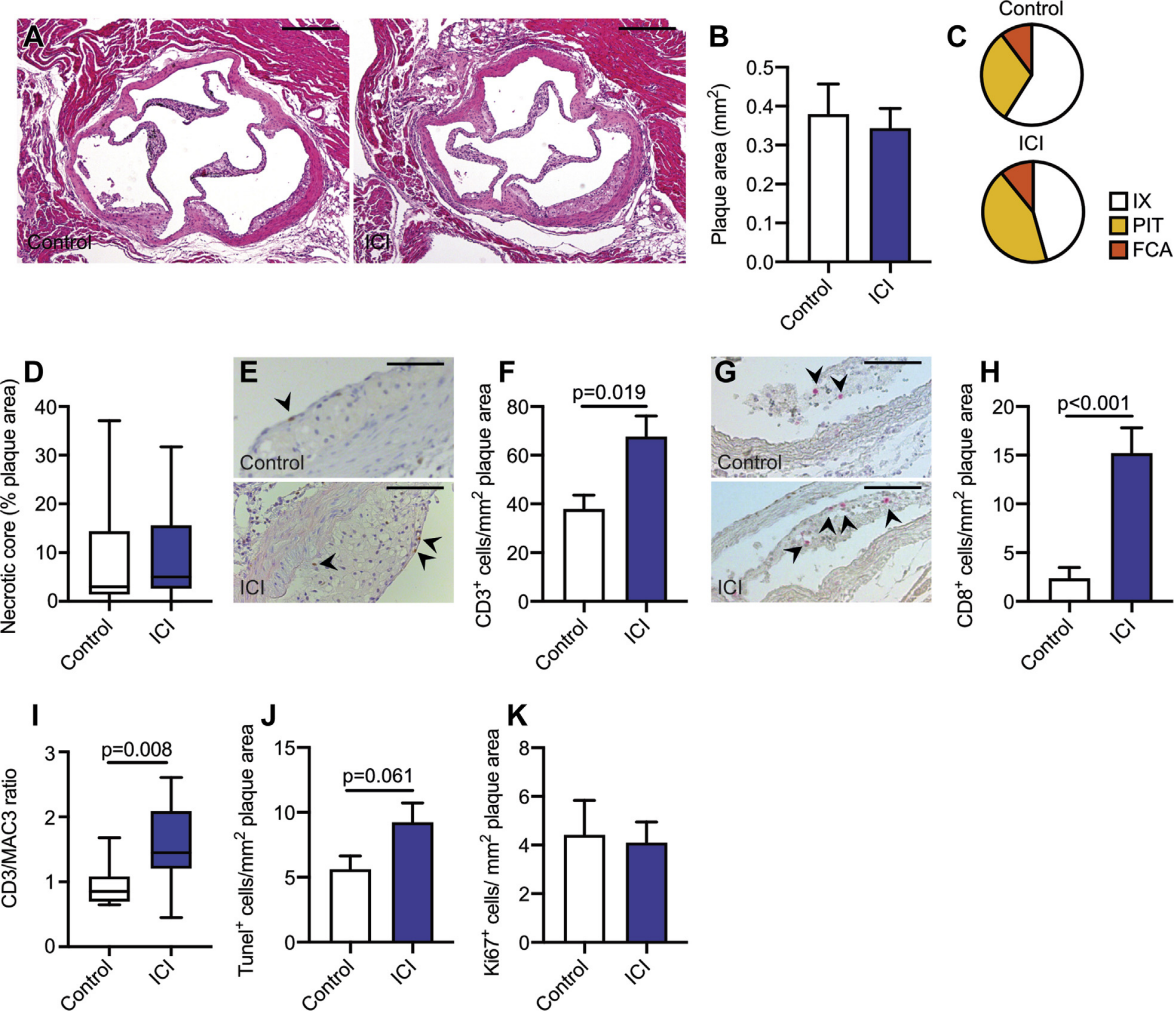
Effects of ICI Treatment on Atherosclerotic Lesions in the Aortic Root of *Ldlr*^*–/–*^ Mice Male *Ldlr*^*–/–*^ mice on high cholesterol diet were treated twice a week with αCTLA-4 and αPD-1 antibodies (n = 15) or isotype control (n = 14) for 5 weeks. The aortic root was analyzed. **(A)** Representative hematoxylin and eosin images of the aortic arch of control and ICI-treated mice. Scale bars represent 200 μm. **(B)** Analysis of plaque size in the aortic root. **(C)** Virmani classification of aortic plaques from control and ICI-treated mice. There was more pathological intimal thickening present in the mice that received ICI treatment. **(D)** Quantification of necrotic core area as a percentage of plaque area. **(E)** Representative images of aortic root CD3^+^ staining in control and ICI-treated mice. Scale bars represent 100 μm. **(F)** Quantification of CD3^+^ cells in the plaque. **(G)** Representative images of aortic root CD8^+^ staining in control and ICI-treated mice. Scale bars represent 100 μm. **(H)** Quantification of CD8^+^ cells in the plaque. **(I)** Ratio of CD3-positive to MAC3-positive areas in the aortic root. Histologic analysis of **(J)** TUNEL^+^ cells and **(K)** Ki67^+^ in the aortic plaque. For all graphs, bars represent mean ± SEM. Abbreviations as in Figures 1 and 4.

Plaques of anti-CTLA4/anti-PD1–treated mice also exhibited a marked increase in CD45^+^ immune cells, especially owing to an increase in the number of CD3^+^ T cells (CD3^+^ T cells per mm^2^ plaque area: control 38.23 ± 10.75 vs. ICI 83.98 ± 12.19; p = 0.014) (Figures 3E and 3F). T cell subset analysis demonstrated a profound increase of CD8^+^ cytotoxic T cells in the plaques of ICI-treated mice (CD8^+^ T cells per mm^2^ plaque area: control 7.46 ± 3.46 vs. ICI 19.94 ± 4.91; p = 0.032) (Figures 3G and 3H, Supplemental Figure 3B). In contrast, plaque macrophage content decreased by 41.9% (Figures 3I and 3J), which resulted in a higher CD3/MAC3 ratio (control 0.92 ± 0.24 vs. ICI 2.80 ± 0.62; p = 0.003) (Figure 3K). This was accompanied by increased number of TUNEL (terminal deoxynucleotidyl transferase dUTP nick end labeling) positive cells (TUNEL^+^ cells per mm^2^ plaque area: control 3.30 ± 1.02 vs. ICI 6.51 ± 1.26; p = 0.039) (Figure 3L). The majority of the TUNEL^+^ cells was also positive for the macrophage marker MAC3, indicating that increased macrophage apoptosis promoted necrotic core formation and subsequent plaque progression (Supplemental Figure 3C). Ki67 expression, smooth muscle cell, and collagen content did not differ between groups (Supplemental Figures 3D and 3E). Similar results were observed in the aortic root (Figure 4, Supplemental Figure 4).

**Figure 5:**
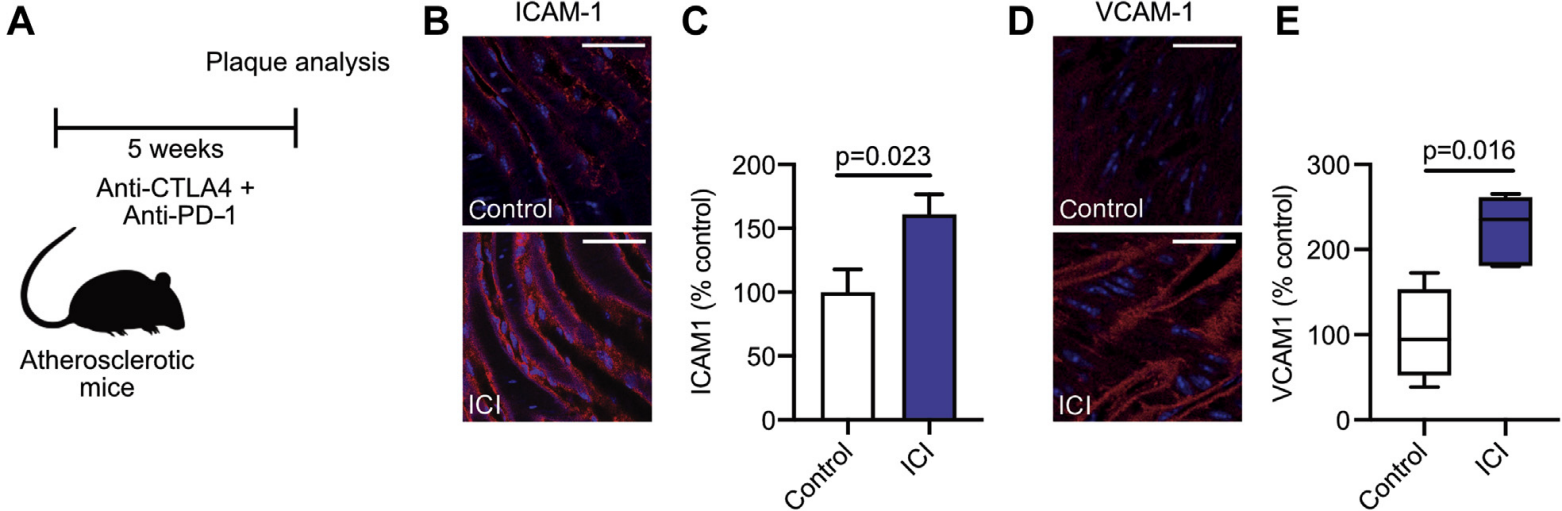
ICI Therapy Increases VCAM Expression in *Ldlr*^*–/–*^ Mice Male *Ldlr*^*–/–*^ mice on high cholesterol diet were treated twice a week with αCTLA-4 and αPD-1 antibodies (n = 15) or isotype control (n = 14) for 5 weeks. Analysis of adhesion molecule expression was performed on endothelium of the abdominal aorta. **(A)** Schematic of experimental design. **(B)** Representative images of intercellular adhesion molecule (ICAM)-1 staining in control and ICI-treated mice. Scale bars represent 20 μm. **(C)** Quantification of ICAM-1 expression. **(D)** Representative images of vascular cell adhesion molecule (VCAM)-1 staining in control and ICI-treated mice. Scale bars represent 20 μm. **(E)** Quantification of VCAM-1 expression. For all graphs, bars represent mean ± SEM. Abbreviations as in Figure 1.

Besides T cell activation, anti-CTLA4/anti-PD1 treatment also induced endothelial activation, as shown by increased expression of intercellular adhesion molecule (ICAM)-1 (% mean fluorescence intensity: control 100.0 ± 17.94 vs. ICI 160.7 ± 15.72; p = 0.023) (Figures 5A to 5C) and vascular cell adhesion molecule (VCAM)-1 (% mean fluorescence intensity: control 100.0 ± 27.61 vs. ICI 223.9 ± 18.26; p = 0.016), on aortic endothelium of the abdominal aorta, which represents the most initial phase of atherosclerosis before immune cell infiltration has commenced (Figures 5D and 5E).

## Discussion

The key observation in our study is that short-term ICI treatment has profound effects on experimental atherosclerosis. We here show that antibody-mediated inhibition of CTLA-4 and PD-1 aggravated plaque inflammation, characterized by a marked increase in cytotoxic CD8^+^ T cells, and increased necrotic core formation, which resulted in the development of more advanced atherosclerosis (Central Illustration).

**Central Illustration:**
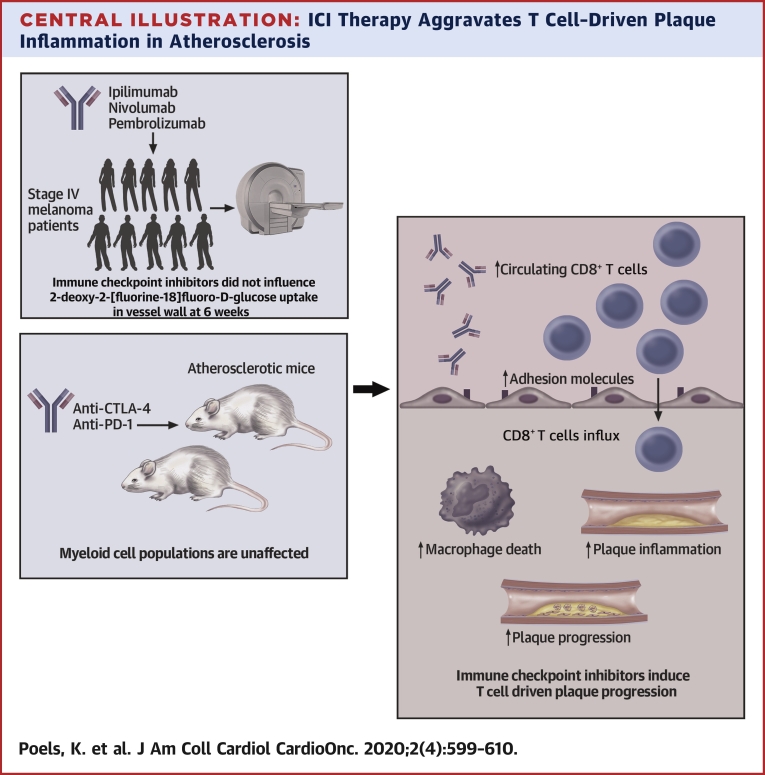
ICI Therapy Aggravates T Cell–Driven Plaque Inflammation in Atherosclerosis Short-term immune checkpoint inhibitor (ICI) therapy does not affect 2-deoxy-2-[fluorine-18]fluoro-D-glucose uptake in vessel wall in stage IV melanoma patients. In hyperlipidemic mice, myeloid cell populations were unaffected upon anti-CTLA-4/anti-PD-1 treatment. However, ICI therapy induced profound T cell activation and CD8^+^ T cell–driven atherosclerotic plaque progression.

Single-cell RNA sequencing and mass cytometry of human atherosclerotic lesions recently demonstrated that T cells are a dominant cell type in plaques and comprise 65% of its total immune cells (23). The frequency of CD8^+^ T cells in the plaques was higher than in the circulation, whereas the reverse was observed for CD4^+^ T cells, reflecting an enrichment of cytotoxic T cells in atherosclerotic lesions (23). T cells within the plaque had a transcriptional profile that reflected activation, cytotoxicity, and T cell exhaustion, in particular in patients with symptomatic atherosclerotic disease, highlighting the detrimental role of cytotoxic T cells in atherogenesis (23). In preclinical studies, adoptive transfer of CD8^+^ T cells in hyperlipidemic lymphocyte-deficient *Rag2*^*–/–*^ mice increased atherosclerotic lesion size and necrotic core formation by promoting granzyme B–, perforin-, and tumor necrosis factor–induced apoptosis of macrophages, smooth muscle cells, and endothelial cells (24). In the present study, we demonstrate that ICI therapy induces an increase in CD8^+^ T cells in the plaque, which is associated with a more detrimental plaque phenotype. The ICI-induced increase in CD8^+^ T cells was accompanied by a reduction in macrophages in the aortic wall, and an increase in the number of apoptotic macrophages. This indicates that these cytotoxic T cells provoke macrophage death, thereby enhancing necrotic core formation and progression toward clinically unfavorable plaques.

One cause of the aberrant infiltration of CD8^+^ T cells in the arterial wall and other nonlymphoid tissues of mice that received ICIs is the increased migratory potential of these cells upon inhibition of CTLA-4 and PD-1 (25,26). A similar phenotype is found in *Ctla4*^*–/–*^ mice, which develop massive lymphocytic infiltrates in various organs (27). Accordingly, CTLA-4 prevents activated T cells to infiltrate peripheral nonlymphoid organs, whereas antibody-mediated inhibition of CTLA-4 increases the motility of tumor infiltrating cytotoxic T cells (26,28). Under hyperlipidemic conditions, T cell specific overexpression of CTLA-4 limits T cell activation and hampers their accumulation in the arterial wall, thereby ameliorating atherosclerosis (29). A comparable phenotype is observed in *Pd1*^*–/–*^*Ldlr*^*–/–*^ mice, which show enhanced infiltration of cytotoxic T cells into the arterial wall (30,31). Genetic deficiency of PD-1 also increased the cytotoxic potential of these cells, which resulted in a marked increase in apoptotic cells, in particular smooth muscle cells and endothelial cells, thereby aggravating atherosclerosis (31). In addition to the enhanced motility of T cells upon immune checkpoint inhibition, the increased expression of the adhesion molecules ICAM-1 and VCAM-1 on the aortic endothelium may have facilitated T cell infiltration into the arterial wall of ICI-treated mice. Taggart et al. (32) demonstrated that antibody-mediated inhibition of CTLA-4 and PD-1 increased the expression of adhesion molecules on tumor endothelium via T cell–derived interferon gamma–driven mechanisms, thereby enhancing lymphocyte migration into the tumor (32). Our data suggest that similar pathways contributed to the increased accumulation of T cells in atherosclerotic plaques of our anti-CTLA-4/anti-PD-1–treated mice.

After migration into the arterial wall, T cells fuel the ongoing inflammatory response by secreting effector molecules, which drive the progression of atherosclerosis (33). In patients with symptomatic atherosclerotic CVD, the majority of T cells display a gene expression profile that is consistent with activation and differentiation (23). Interestingly, several clusters of CD4^+^ and CD8^+^ T cells within the human plaque express high levels of PD-1, which is one of the characteristics of an exhausted T cell phenotype, possibly due to chronic low-grade inflammation (23). Whether inhibition of PD-1 induces (re)activation of these exhausted T cell populations in the plaque is currently unknown, but this potential mechanism may have contributed to the increased progression of atherosclerosis in our study.

Encouragingly, in a very small retrospective analysis, we did not observe an increase in ^18^F-FDG uptake in the large arteries, bone marrow, or spleen of patients with advanced melanoma that were treated with ICIs for a short term. Previous studies, which used ex vivo micro-PET to characterize ^18^F-FDG uptake in specific regions of endarterectomy species, demonstrated that macrophages and foam cells are responsible for the majority of ^18^F-FDG uptake in the atherosclerotic plaque (19). Therefore, ^18^F-FDG PET cannot be exploited to detect T cell–driven plaque inflammation. Alternative techniques, such as immune PET, may be more suitable but are currently not routinely available. Our findings thus indicate that short-term ICI therapy does not affect monocyte- or macrophage-driven inflammation in the arterial wall in mice and men, which is in accordance with studies in *Pd1*^*–/–*^*/Ldlr*^*–/–*^ and *Ctla4-tg-Apoe*^*–/–*^ mice (34, 35, 36). Whether long-term ICI therapy affects myeloid-driven inflammatory responses in the arterial wall is unknown. Nevertheless, our data suggest that even short-term treatment aggravates plaque inflammation. Whether these immune checkpoint inhibitor–induced alterations persist after cessation of the therapy and how they affect atherogenesis in the long-term is currently unknown.

Clinical data on ICI-induced atherosclerosis-related adverse events are still scarce, as the majority of the clinical trials excluded both patients with previous CVD and the elderly, who often have subclinical atherosclerosis. Moreover, clinical complications of atherosclerosis develop gradually over many years or even decades. As ICI therapy was clinically implemented only in the past years, the potential long-term cardiovascular side effects are still unknown. Nevertheless, the number of reports on atherosclerosis-related vascular events in ICI-treated patients is increasing. For example, a meta-analysis of 22 clinical trials showed 3% of the patients that received anti-PD(L)-1 therapy developed a myocardial infarction or ischemic stroke (37). Additionally, 1% of the ICI-treated patients with advanced lung cancer developed a myocardial infarction or ischemic stroke within 6 months after initiation of therapy, suggesting that the event resulted from progression of existing atherosclerotic plaques and not on the formation of new lesions, which typically take years to decades to develop (17). Interestingly, a recent autopsy study in 11 cancer patients, who died from noncardiovascular causes, demonstrated that ICI therapy increased the T cell-to-macrophage ratio in coronary atherosclerotic lesions, reflecting a predominantly T cell–driven inflammatory response that is comparable to our findings in anti-CTLA4/anti-PD1–treated hyperlipidemic mice (38). Although an increased CD3/macrophage ratio is associated with symptomatic atherosclerotic CVD, large clinical studies with long-term follow-up are required to unequivocally determine the clinical relevance of checkpoint inhibitor–induced plaque inflammation.

Treatment with ICIs is standard of care for an increasing number of cancer types and is also applied in patients with an elevated risk for atherosclerotic CVD, such as the elderly and patients who receive radiotherapy or chemotherapy, which may also affect atherosclerosis. Specifically, for this heterogeneous population, more extensive clinical studies are necessary to elucidate the effects of ICI therapy on the development of cardiovascular events. Until then, optimal cardiovascular risk management should be implemented, especially as these adverse events severely compromise the quality of life and mortality rates of cancer patients (40).

### Study limitations

Our human study was performed in a small cohort of patients and focused on the short-term effects of ICI therapy, which is in contrast to the clinical studies discussed previously that focused on atherosclerotic CVD in patients during a 3-year period (17). Furthermore, our patients did not have a history of CVD, and it is possible that the detrimental vascular effects of immune checkpoint inhibition, which we observed in our preclinical study, are more prominent in patients with subclinical atherosclerosis or a history of CVD. More extensive studies are required to elucidate the long-term effects of inhibition of inhibitory immune checkpoints on atherosclerosis in humans, especially as atherosclerosis-related complications may develop over years or decades. It would also be relevant to include other patient populations that more frequently have subclinical atherosclerosis, such as the elderly, patients with non–small cell lung cancer, and ICI-treated patients who received radio- or chemotherapy, which may also affect atherosclerosis (39). As our study population was too small to analyze these potential effects, these important topics should be addressed in future studies. Another limitation of our study is the use of a tumor-free atherosclerosis model. As atherosclerosis and cancer share many pathophysiological pathways, including inflammation, the use of a tumor-bearing atherosclerosis model would have increased the translation potential of our study.

## Conclusions

Short-term ICI therapy did not affect myeloid-driven inflammation in the large arteries of patients with advanced melanoma. However, antibody-mediated inhibition of CTLA-4 and PD-1 aggravated T cell–driven inflammation in atherosclerotic plaques of hyperlipidemic mice and provoked the progression of atherosclerosis toward a clinically unfavorable plaque phenotype. Based on these data, we feel that additional clinical studies are required to elucidate the long-term effects of ICI therapy on atherosclerotic CVD in order to develop evidence-based strategies to prevent potentially serious cardiovascular complications in cancer patients and long-term cancer survivors.

Perspectives **COMPETENCY IN MEDICAL KNOWLEDGE:** Short-term ICI therapy in hyperlipidemic mice aggravates T cell–driven inflammation in atherosclerotic lesions, which promotes the progression toward clinically unfavorable plaque.

**TRANSLATIONAL OUTLOOK:** This study suggests that short-term ICI therapy affects atherosclerosis. Long-term clinical studies are required to elucidate the effects of ICIs on atherosclerosis in cancer patients, especially as atherosclerotic CVD may develop over years or decades.

## Author Disclosures

This study was supported by the Netherlands Heart Institute (Young@heart grant to Dr. Seijkens), the Dutch Heart Foundation (Dr. Dekker Physician-in-specialty-training grant to Dr. Seijkens), the Netherlands Organization for Scientific Research (VICI grant 016.130.676 to Dr. Lutgens, VICI grant 91818622 to Dr. Mulder), the European Union (H2020-PHC-2015-667673, REPROGRAM to Dr. Lutgens), the European Research Council (ERC consolidator grant CD40-INN 681492 to Dr. Lutgens), and the German Science Foundation (CRC1123, project A5 to Dr. Lutgens). This work was also supported by the Netherlands CardioVascular Research Initiative: the Dutch Heart Foundation, Dutch Federation of University Medical Centres, the Netherlands Organisation for Health Research and Development, and the Royal Netherlands Academy of Sciences for the GENIUS-II project “Generating the best evidence-based pharmaceutical targets for atherosclerosis,” the National Institutes of Health (grants R01 CA220234, R01 HL144072, and P01 HL131478 to Dr. Mulder), and the American Heart Association (grant 19PRE34380423 to Dr. van Leent). Dr. Marabelle has served on scientific advisory boards and provided consulting services for Bristol Myers Squibb, Merck Sharp & Dohme, Pfizer, AstraZeneca, Sanofi, and Roche. All other authors have reported that they have no relationships relevant to the contents of this paper to disclose.
