# Supplementary material for: Immune Checkpoint Inhibitor Therapy Aggravates T Cell–Driven Plaque Inflammation in Atherosclerosis
Source: JACC CardioOncol. 2020 Oct 6;2(4):599–610. doi: 10.1016/j.jaccao.2020.08.007 (PMC8352210; doi:10.1016/j.jaccao.2020.08.007)
Supplement: Supplemental Appendix and Supplemental Figures 1–4 [file mmc1.docx]

**SUPPLEMENTAL APPENDIX**

**Materials & methods**

***^18^F FDG PET/CT imaging of patients***

Image acquisition using FDG-PET/CT was performed without intravenous contrast at baseline and 6 weeks after initiation of immune checkpoint inhibitor (ICI) therapy. Site ‘Antony’ performed seven scans using a Philips Gemini TFTOF 16 scanner with 120 kVp, 5mm slice thickness, B filter type, B convolution kernel, with an unknown pitch and a PET pixel spacing of 4. ^18^F-fluorodeoxyglucose (^18^F-FDG) dose at site ‘A’ ranged from 3.23-3.88 MBq/kg and circulation time ranged from 55-114 minutes. Site ‘Roussy’ performed 9 scans using a GE Discovery 690 scanner with a range of 100-140 kVp, 2.5mm slice thickness, Body filter type, Standard convolution kernel, with a pitch of 1.75 and a PET pixel spacing of 2.73. ^18^F-FDG dose at site ‘R’ ranged from 3.38-3.56 MBq/kg and circulation time ranged from 56-80 minutes. Site ‘Marie Lannelongue’ performed two scans using a GE Discovery 690 scanner with 120 kVp, 3.75mm slice thickness, Body filter type, Standard convolution kernel, with a pitch of 1.75 and a PET pixel spacing of 2.73. ^18^F-FDG dose at site ‘ML’ ranged from 2.67-2.71 MBq/kg and circulation time ranged from 54-107 minutes. Site ‘CHU Amiens Picardie’ performed one scan using a Siemens Biograph CT/1080 PET scanner with 110 kVp, 2.5mm slice thickness, ‘1’ filter type, B31S convolution kernel, with an unknown pitch and a PET pixel spacing of 4.06. ^18^F-FDG dose at site ‘CAP’ was 4.51 MBq/kg and circulation time of 69 minutes. Site ‘Medical Nuclear Clinique du Europe’ performed one scan using a GE Optima 560 scanner with 120 kVp, 2.5mm slice thickness, Body filter type, Standard convolution kernel, with a pitch of 1.375 and a PET pixel spacing of 2.73. ^18^F-FDG dose at site ‘MNCduE’ was 3.18 MBq/kg and circulation time of 65 minutes.

***^18^F FDG PET/CT imaging of atherosclerotic mice***

15 week old *Apoe^-/-^* mice were anesthetized with ketamine (100 mg/kg) and xylazine (6 mg/kg) via intraperitoneal injection and 1% isoflurane (Baxter Healthcare, Deerfield, IL)/oxygen gas mixture. Animals were injected with 266.7 ± 18.3 μCi ^18^F-FDG via the tail vein. 60 minutes after injection, a scan was performed using a Mediso nanoScan PET/CT (Mediso, Budapest, Hungary). A whole body CT scan was performed first (energy 50kVp, current 180 μAs, isotropic voxel size at 0.25 mm), after which a 30-minute PET scan was performed. Reconstruction was performed using the TeraTomo 3D reconstruction algorithm, with attenuation correction, with the use of Mediso Nucline software. An energy window between 400 – 600 keV was used to filter coincidences. The voxel size was isotropic with 0.4 mm width and the reconstruction was applied for four full iterations, six subsets per iteration.

***^18^F FDG PET/CT analysis***

The aorta and bilateral carotid arteries were assessed by measuring the mean and maximum Standardized Uptake Value (SUV) by drawing regions of interest (ROIs) along the outer boundary of each vessel in axial section. The carotid arteries were analyzed separately. Mean and maximum SUVs were recorded in each slice: SUVmeanSL and SUVmaxSL. In addition, SUVs of the arteries are normalized to the background uptake. Background uptake was measured within the blood pool within veins (i.e., jugular veins, superior vena cava, inferior vena cava). For background measurements an oval drawing tool was used to find the lowest fused image SUVmean within 10 sample slices. This method allowed for calculation of Target-to-Background ratios (TBR), both mean and maximum, by dividing SUVmeanSL and SUVmaxSL by the average of SUVmean over all 10 background slices. A similar method was employed to assess the spleen and bone marrow. The spleen was traced in its entirety and the bone marrow was assessed in the thoracolumbar spine. Background for the spleen and bone marrow was acquired in both the inferior vena cava and in the erector spinae muscles to allow for additional comparison. Image analysis of murine PET/CT scans was performed in analogy with the analysis of the patient PET/CT scans. However, here the thoracolumbar spine was traced on the sagittal plane and the spleen was traced on the coronal plane.

***Gamma counting***

After completion of the PET/CT scan, mice (n=8) were euthanized and perfused with 20 mL PBS. Aortas were dissected from the root until the aortic bifurcation and surrounding fat was removed. Femurs and spleens were harvested. Tissues were weighted and ^18^F-FDG content was using a Wizard2 2480 automatic gamma counter (Perkin Elmer). ^18^F-FDG uptake is expressed as % of injected dose per gram of tissue (%ID/g).

***Animal experiments***

Male *Ldlr^-/-^* mice were bred and housed at the local animal facility and were fed a 0.15% cholesterol diet *ad libitum* from the age of six weeks. Starting at 12 weeks of age, mice were injected IP twice a week for five weeks with anti-CTLA4 (BE0164, BioXcell, West Lebanon, NH, USA) and anti-PD-1 (BE0146, BioXcell) antibodies (200 µg) (n=15) or isotype (IgG2b and IgG2a) control antibodies (n=14) (BE0086 and BE0089 (200 µg) BioXcell). For FDG PET/CT studies, female *Apoe^-/-^* mice (n=16) were fed a 0.15% cholesterol diet *ad libitum*. Animals were then treated twice a week with anti-CTLA-4 (BE0164, BioXcell) and anti-PD-1 (BE0146, BioXcell) antibodies (200 µg) (n=8) or PBS (n=8) for 4 weeks. All experiments were approved by the animal welfare committee of Icahn School of Medicine at Mount Sinai or Amsterdam University Medical Center (protocol: DBC-17-1666-1-09).

***Flow cytometry***

Spleen and lymph nodes were homogenized. Blood and spleen samples were subjected to red blood cell lysis. Aortas were dissected from the root until the aortic bifurcation and gently cleaned from surrounding fat. Aortas were digested using an enzymatic digestion solution containing Liberase TH (4 U/mL) (Roche), deoxyribonuclease (DNase) I (40 U/mL) (Sigma-Aldrich), and hyaluronidase (60 U/mL) (Sigma-Aldrich) in PBS at 37°C for 60 minutes. The cells were stained with fluorescently labeled surface antibodies (CD45, CD3, CD4, CD8, F4/80, Ly6C, Ly6G, CD44, CD62L, CD19, CD11c, NK1.1, CD11b, CD90.2, Ter119, CD49b, CD45R; from BD Biosciences, eBioscience and BioLegend). For intracellular staining, the cells were fixed and permeabilized with fixation/permeabilization buffer (eBioscience, Waltham, MA, USA) and stained with fluorescent antibodies against Foxp3 (BioLegend, San Diego, CA, USA). Flow cytometric analysis was performed on a BD Canto II or BD LSR Fortessa (BD Biosciences). All flow cytometry stainings and panels have been evaluated and optimized in pilot experiments before being used in this study. FMOs and isotype controls were included in these analyses and are available on request.

***Histology***

Immunohistochemistry was performed for CD3 (AbD Serotec, Veenendaal, the Netherlands), CD8 (AbD Serotec), MAC3 (BD Pharmingen, San Diego, CA, USA), α-smooth muscle actin (αSMA) (Sigma, Zwijndrecht, the Netherlands), and Ki67 (Abcam, Cambridge, United Kingdom). To detect apoptotic cells, sections were using the In Situ Cell Death Detection Kit (Roche, Zwijndrecht, the Netherlands). Collagen was detected by sirius red staining. Other organs were fixed in 4% PFA and embedded in paraffin. Sections were stained with hematoxylin and eosin or CD3 as mentioned above. Morphometric analyses were performed on a Leica DM3000 microscope with a DFC 295 camera and Adobe Photoshop CS6, Image J or Las4.0 software (Leica, Amsterdam, the Netherlands).

***Confocal microscopy***

The abdominal aorta was isolated, fixed in 4% PFA for 12 minutes and stained for ICAM1 (Abcam) and VCAM1 (Abcam) in combination with DAPI. *En face* sections were obtained on a Leica SP8 confocal microscope and quantification was performed with Image J.

***Statistics***

Data are depicted as mean ± standard error of the mean (SEM). Normal distribution was analyzed with a D’Agostino Pearson omnibus normality test, while between group differences were compared using an unpaired Student's t-test or Mann Whitney test as appropriate based on variable distribution. Paired t-tests were used to analyze differences in patient ^18^F-FDG PET signal at baseline and post treatment. A chi-square test was used to analyze the contingency of the Virmani classifications. For all analyses GraphPad Prism 5.0 software (GraphPad Software Inc., San Diego, CA, USA) was used. P-values < 0.05 were considered significant.

**Supplemental Figure 1.** **^18^F-FDG PET based analysis of immune checkpoint inhibitor (ICI) therapy in *Apoe^-/-^* mice.** *Apoe^-/-^* mice (n=8) were treated with ICI or PBS for four weeks and subjected to ^18^F-FDG PET/CT imaging, before or after treatment. (**A**) Flow cytometry analysis of Ly6C^lo^ monocytes and neutrophils in the aorta. (**B**) *Ex vivo* quantification of ^18^F-FDG accumulation in the femur and spleen. For all graphs, bars represent mean +/- SEM.

**
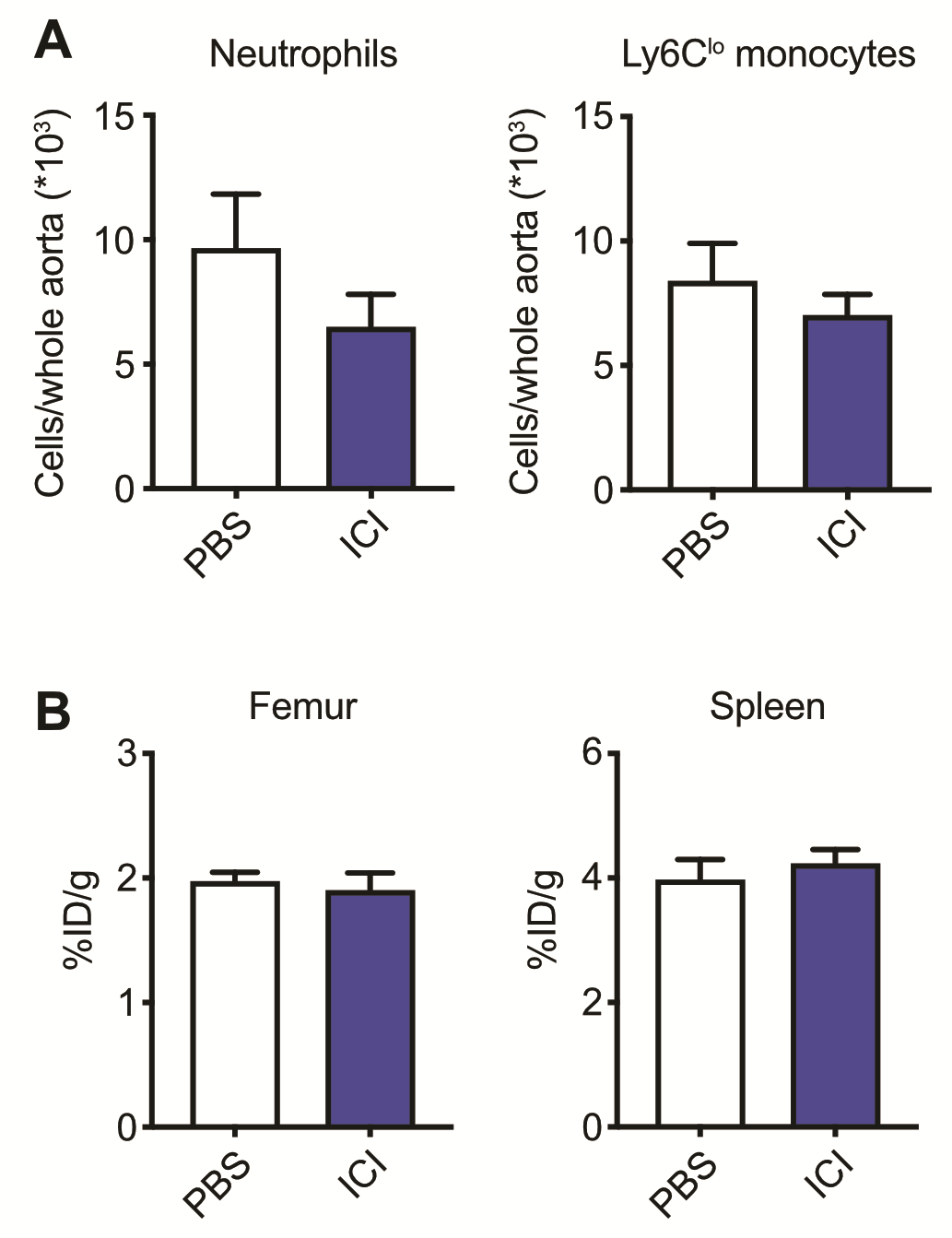
**

**Supplemental Figure 2. ICI therapy induces an activated T-cell profile in the spleen and blood of *Ldlr^-/-^* mice.** Male *Ldlr^-/-^* mice first received 6 weeks of 0.15% high cholesterol diet, after which they were treated biweekly with αCTLA-4 and αPD-1 antibodies (n=15) or isotype control (n=14) for five weeks. (**A**) Flow cytometric analyses of myeloid populations in the spleen. (**B**) Flow cytometric analyses of lymphoid populations in the blood. For all graphs, bars represent mean +/- SEM.

**
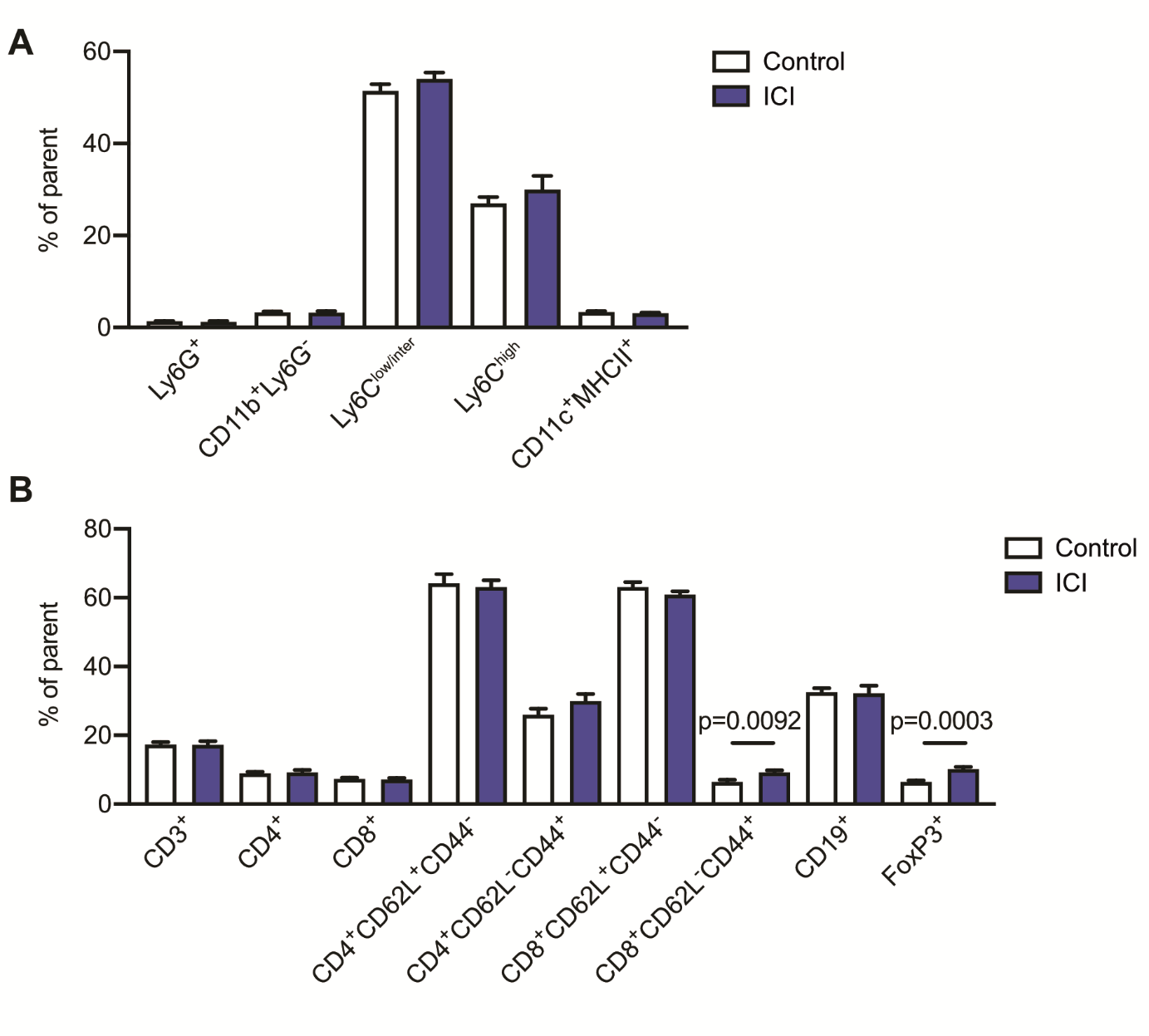
**

**Supplemental Figure 3. Effects of ICI treatment on atherosclerotic lesions in the aortic arch of *Ldlr^-/-^* mice.** Male *Ldlr^-/-^* mice on high cholesterol diet were treated biweekly with αCTLA4 and αPD1 antibodies (n=15) or isotype control (n=14) for five weeks. The aortic arch was analyzed. (**A**) Contingency analysis of the Virmani classification. (**B**) Quantification of CD4^+^ cells in the plaque. (**C**) Quantification of MAC3^+^ Tunel^+^ cells as a percentage of Tunel^+^ cells, based on a co-staining assay (**D**) Quantification of Ki67^+^ cells in the plaque. (**E**) Histologic analysis of αSMA and collagen in the aortic arch. For all graphs, bars represent mean +/- SEM.

**
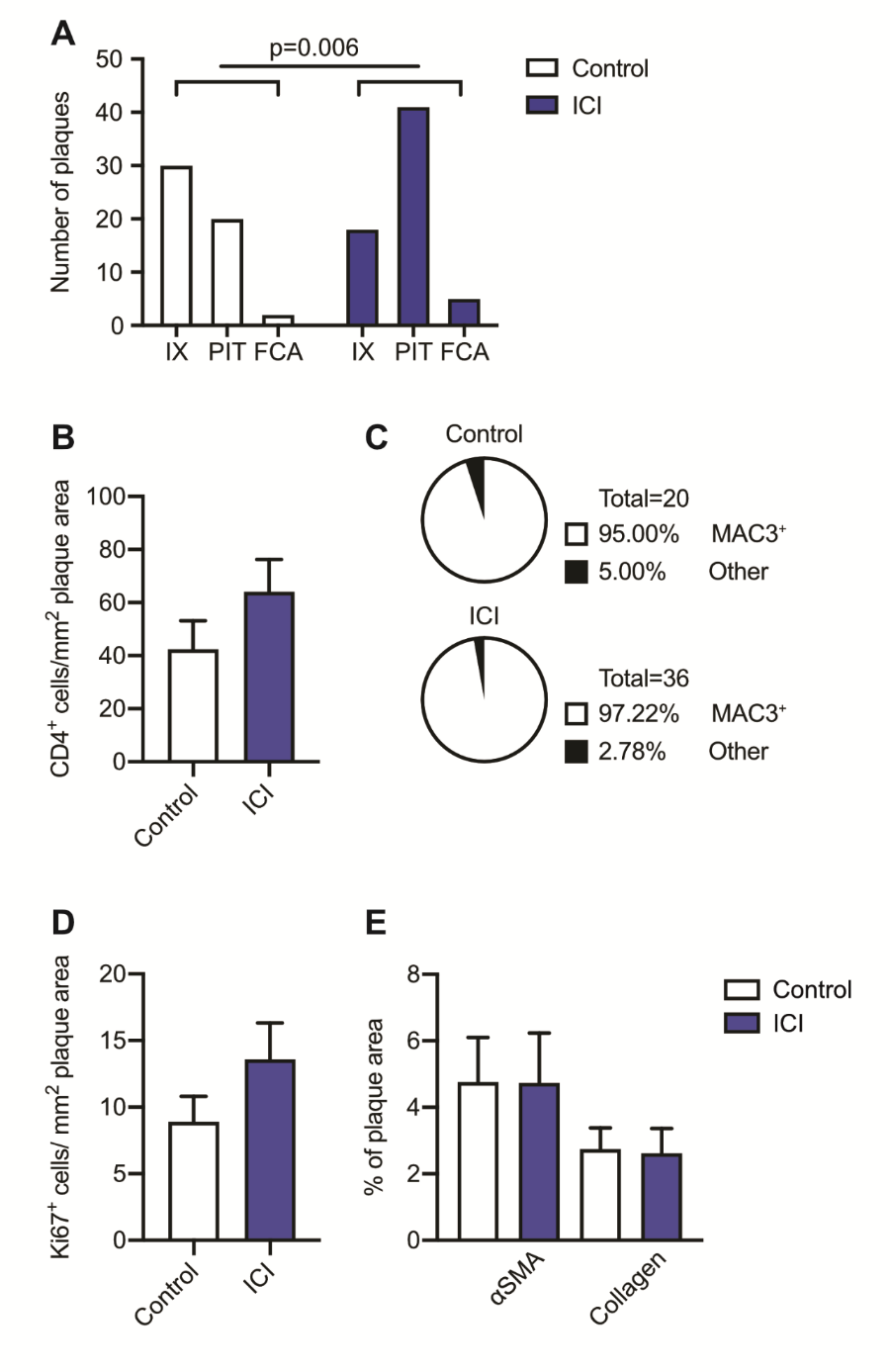
**

**Supplemental Figure 4. Effects of ICI treatment on atherosclerotic lesions in the aortic root of *Ldlr^-/-^* mice.** Male *Ldlr^-/-^* mice on high cholesterol diet were treated biweekly with αCTLA4 and αPD1 antibodies (n=15) or isotype control (n=14) for five weeks. The aortic root was analyzed. (**A**) Contingency analysis of the Virmani classification (**B**) Quantification of CD4^+^ cells in the plaque. (**C**) Quantification of MAC3^+^ areas as a percentage of total plaque area. (**D**) Histologic analysis of αSMA and collagen in the aortic root. For all graphs, bars represent mean +/- SEM.

**
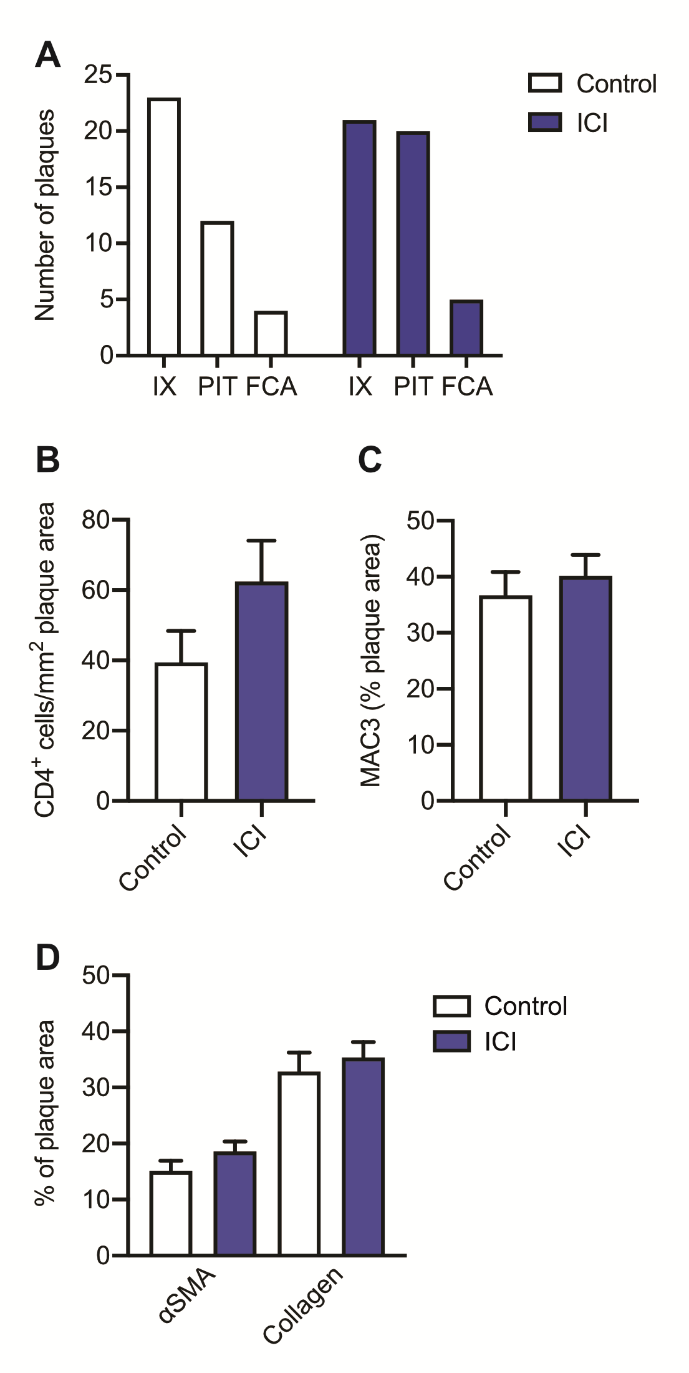
**
